# The 1470 nm diode laser with an intralesional fiber device: a proposed solution for the treatment of inflamed and infected keloids

**DOI:** 10.1186/s41038-019-0143-6

**Published:** 2019-02-15

**Authors:** Ke Li, Fabio Nicoli, Wen Jing Xi, Zheng Zhang, Chunxiao Cui, Ahmed Al-Mousawi, Alberto Balzani, Yun Tong, Yixin Zhang

**Affiliations:** 1 Department of Plastic and Reconstructive Surgery, Shanghai Ninth People’s Hospital, Shanghai JiaoTong University School of Medicine, 639 Zhi Zao Ju Road, Shanghai, 200011 China; 2 Department of Plastic and Reconstructive Surgery, University of Rome “Tor Vergata”, Rome, Italy; 3 Department of Plastic and Reconstructive Surgery, Newcastle Hospitals NHS Foundation Trust, Newcastle upon Tyne, UK; 4 Department of Medical Cosmetology Surgery, Jinhua People’s Hospital, Jinhua, China

**Keywords:** Hypertrophic scars, Keloid scars, 1470 nm, Diode laser, Fiber laser

## Abstract

**Background:**

Keloids are the result of abnormal wound healing and often are subject to infections and recurrent inflammation. We present a study conducted with a 1470 nm diode laser using an intralesional optical fiber device for the treatment of inflamed keloid scars. We evaluate its efficacy as a novel alternative method to decrease keloid infection and inflammation.

**Methods:**

The patients who underwent 1470 nm laser treatment from February 2016 to February 2018 at the plastic and reconstructive surgery department of the Shanghai Ninth People’s Hospital Affiliated to Shanghai Jiao Tong University with keloid accompanying serious local infection and fester were included. Patients took curative effect evaluation before and 1 year after the treatment. The test items included infection frequency in each year; pain, by visual analogue scale (VAS); itch, using VAS; quality of life (QOL), using QOL scale; and blood supply, using PeriCam PSI.

**Results:**

A total of 19 patients (mean age 35.21 years, range 11–66) with history of inflamed keloids with episodes of infection or abscess were enrolled. Patients underwent to a 1470 nm laser therapy for average of 1.16 times. After treatment, infection frequency and blood supply in keloids were reduced (*p* < 0.001). Pain, itching, and QOL were improved (*p* < 0.001).

**Conclusion:**

The present study shows that 1470 nm fiber laser treatment could improve inflamed keloids fairly well by decreasing inflammation, and a relative stabilization of collagen composition. Therefore, it is an effective minimally invasive scar therapy, but further studies are essential to confirm the present results.

## Background

Keloid scars are the consequences of several skin disorders such as laceration, burns, surgery or abscess, acne, tattoos, injections, and ear-piercing. These pathological scars are characterized by continuous inflammation and histologically, fibroblasts proliferation, newly formed blood vessels and collagen deposition. By definition hypertrophic scars are confined to the borders of the original wound while keloids overgrowth beyond the borders and tend to recur [1, 2]. The etiology of keloids is still unclear, but they occur after dermal injuries in genetically predisposed individuals and can cause both physical and psychological discomfort for the affected people. Among them, deep burns are reported to be the main cause of keloids [3]. Other hypotheses about the origin of keloids are based on keratin stimulation, wound tension, sensitization to sebum, viral, or fungal infections [4].

In general, patients seek treatment to relieve pain, itching, and restriction of functions, but esthetic improvement usually represents the main or sole motive for the treatment [5].

Furthermore, the keloids can be characterized by prolonged inflammation or over infection that can complicate the course of the wound healing and make a difficult decision about the treatment of choice [6].

Due to the histopathological variety of keloid scars and unknown etiology, several treatments have been described with alternate results. Non-invasive therapies such as silicone sheet, gel application, and pressure garments may result in easily application and management but with long lasting procedure and sometimes poor outcomes [6, 7]. Topical and intralesional injections with corticosteroids, interferon, bleomycin, and 5-Fluorouracil (5-FU) may have better and faster outcomes but can result in more pains and lead to infections. [7, 8]

Minimally invasive or non-invasive methods such as cryotherapy, laser therapy, laser therapy with drug delivery, and radiotherapy have demonstrated good outcomes for soft and thin scars. However, traditional laser diffusion also has poor efficacy and penetrance for harder texture and thicker scars. Moreover, topical medications scarcely penetrate, and drug injection diffusion is blocked by fibrotic tissue, necessitating multiple and repeated treatment, with often limited efficacy [7–9]. These treatments can also irritate more the scar tissue provoking a worsen wound healing. Surgical excision for inflamed keloids remains an option but unfortunately long-term results can be poor due to over infection, wound dehiscence, prolonged wound healing, and high risk of scar recurrence and hyperplasia associated with worsening of the wound and poor esthetic outcome. Currently, few minimally invasive techniques resulted really effective to reduce postoperative scarring, decrease inflammation, and improve scar texture of keloids [5].

The authors introduced an alternative minimally invasive laser and have investigated the effects on inflamed keloids using the 1470 nm diode laser with intralesional fiber [10–12]. The bare optical fiber can be used to overcome some inherent limitations when used to deliver energy from an infrared laser. The 1470 nm wavelength is absorbed by water and hemoglobin, particularly deoxyhemoglobin, penetrating 2–3 mm in depth [12–15].

The 1470 nm diode laser and fiber system therefore causes localized heating within a narrow range. Local tissue moisture rapidly vaporizes and cells undergo lysis, necrosis, and solidification, resulting in tissue ablation. Additionally, the 1470 nm laser functions to coagulate blood vessels, reducing blood supply and local tissue vascularization [12–17].

The aim of this study is to evaluate the efficacy and safety of 1470 nm fiber laser for the treatment of inflamed or infected keloids, which may represent an alternative treatment for this pathological condition.

## Methods

### Inclusion criteria

This was a prospective study to evaluate the efficacy and safety of a fractional non-ablative diode laser and fiber to treat patients with inflamed keloids associated with infections, abscess, or suppurative manifestations. Various anatomical regions were involved in the study with multifactorial causes as well as trauma, burns, acne, or surgery. Nineteen consecutive patients were enrolled between February 2016 and February 2018 and treated at the Ninth People’s Hospital in Shanghai. Patients were treated with one to three laser sessions.

### Exclusion criteria

Patients who had undergone scar therapy within 1 year, had abnormal cardiopulmonary function, received immunosuppressive drugs or had autoimmune disorders or uncontrolled diabetes, and those who were not able to follow the survey were excluded.

### Follow-up plan

Tests were repeated three times in each case, and the average value was used. Patients were resting for 10 min before examination; all tests were performed in the same room, with the room temperature maintained at 23–25 °C. The scars were clinically evaluated by three experienced independent physicians pre-operatively and at 1 year after treatment.

### Subjective evaluation

Pain and itch were assessed using Visual Analogue Scale (VAS) with 100 grades: 100 represented extreme pain or itch and 0 represented no pain or itch [18]. VAS is a graphic tool with a 100-mm horizontal line with the left end marked as “no symptom” and the right end marked as “worst imaginable symptom.”

A questionnaire was fulfilled to investigate the impact of the scar on quality of life (QOL)[19].

### Objective evaluation

The physicians also recorded the frequency of infection. A standard frontally oriented photograph was taken at 3, 6-month, and 1 year follow-up clinic.

The features of each scar were objectively evaluated measuring the changes of blood supply. The PeriCam PSI System® (Perimed, Järfälla, Sweden) blood perfusion imager was applied to assess changes of the blood supply within the scar. The PeriCam PSI System® is a blood perfusion imager 70-mW system based on Laser Speckle Contrast Analysis (LASCA) technology utilizing a laser wavelength of 785 nm [20].

### Laser technique

This was a prospective case series conducted to evaluate the safety and efficacy of fractional non-ablative laser LASEmaR® 1500 (EUFOTON, Trieste, Italy) as an alternative treatment of inflamed or infected keloids. It uses the semiconductor gallium arsenide (GaAs) to emit 1470 nm wavelengths of light, output to a fiber through a diode with an optical fiber diameter of 300 μm. Power output was adjusted according to scar hardness in order to penetrate the scar with minimal resistance, generally starting from 3 W and gradually increasing to a maximum power output of 12 W if necessary, with a maximum fluence 999.9 KJ/cm^2^. The laser was initiated and the optical fiber used to penetrate and deliver treatment within the scar. Entry points were made around the scar, and the distance between the adjacent entry points was 4–6 mm. The procedure was repeated once every 1 to 2 months until a satisfactory effect was achieved in agreement between the clinician and patient.

### Ethics approval and consent to participate

The study was conducted in accordance with the guidelines of the Declaration of Helsinki stated in 1964. The study protocol was agreed by the local ethics committee of the Jiao Tong University-Ninth People’s Hospital of Shanghai. Every patient signed written informed consent.

### Statistical analysis

The paired *t* test was used to compare the changes of scar blood supply before and after 1470 nm laser treatment. Subjective assessment before and after 1470 nm laser treatment were compared by Wilcoxon rank-sum test. All data analyses were conducted using SPSS version 19.0 with statistical significance set at *p* < 0.05.

## Results

### Patient characteristics

A total of 19 patients (mean age 35.2 ± 16.09 years, range 11–66) with inflamed keloids were enrolled in the study. Eleven patients were male and 8 patients were female. Locations of the scars were chest (12 patients), perineum (4 patients), face (2 patients), and neck (1 patient). All patients described their ethnicity as Asian. The average scar size was 32.47 ± 20.87 cm^2^. The etiology of scars was furuncle (14 patients), burns (3 patients), acne scarring (1 patient), and herpes zoster infection scarring (1 patient). The average number of treatments was 1.16 ± 0.37 (range 1–3). The patients were affected by the scars for a mean period of 5.63 ± 1.98 years, and the lesions were complicated by an infection for 2.63 ± 1.07 years (Table 1).

**Table 1:** Demographic and scar characteristics of study cohort

Characteristic	Value (Mean ± SD)
Age	35.21 ± 16.09 years
Sex	
Female	8
Male	11
Period with keloid	5.63 ± 1.98 years
Period with keloid complicated by infection	2.63 ± 1.07 years
Keloid surface	32.47 ± 20.87 cm^2^
Treatment frequency	1.16 ± 0.37 times
Etiology	
Burn	3
Furuncle	14
Acne	1
Herpes zoster virus infection	1
Fitzpatrick skin type	
II	2
III	12
IV	5

### Objective evaluation

**Fig. 1:**
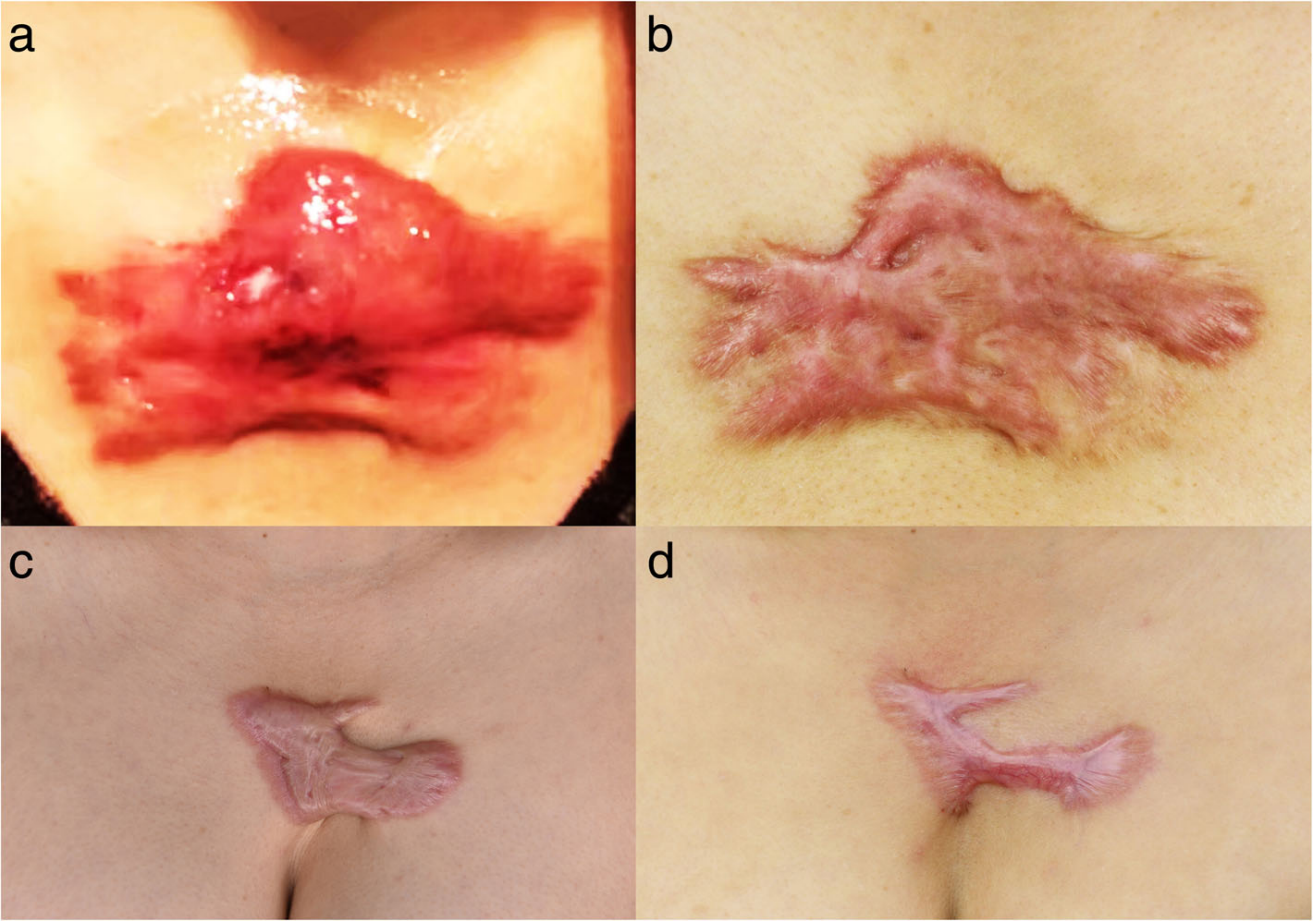
Photographs of cases. A 37-year-old female suffered by inflamed keloid with recurrent episodes of infections 6 times per year (**a**), and 1-year after laser treatment, she did not present any further infection (**b**). A 45-year-old female had an inflamed keloid with 3 times events of infection per year (**c**), and  1-year after laser treatment, no further episodes of infection were registered (**d**)

**Fig. 2:**
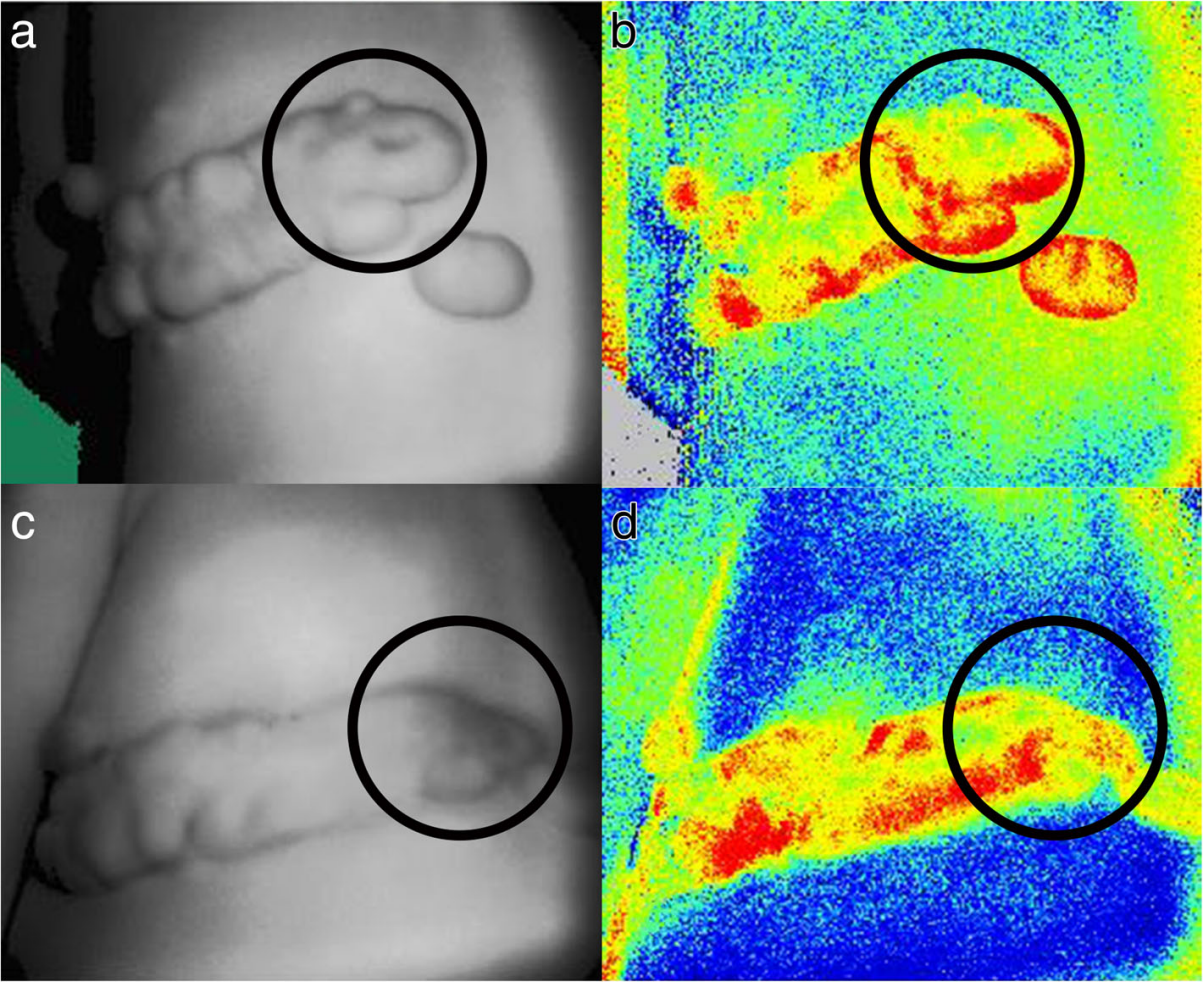
PeriCam PSI blood perfusion imager showing the reduction of blood supply in keloid after 1470 nm laser treatment. A 12-year-old boy had recurrent infections on his keloid 6 times per year (**a**, **b** before treatment). After 1-year laser treatment, no further infections were recorded, and the keloid blood supply reduced to 8.21% (**c**, **d**). The infected area is highlighted in black circle

**Fig. 3:**
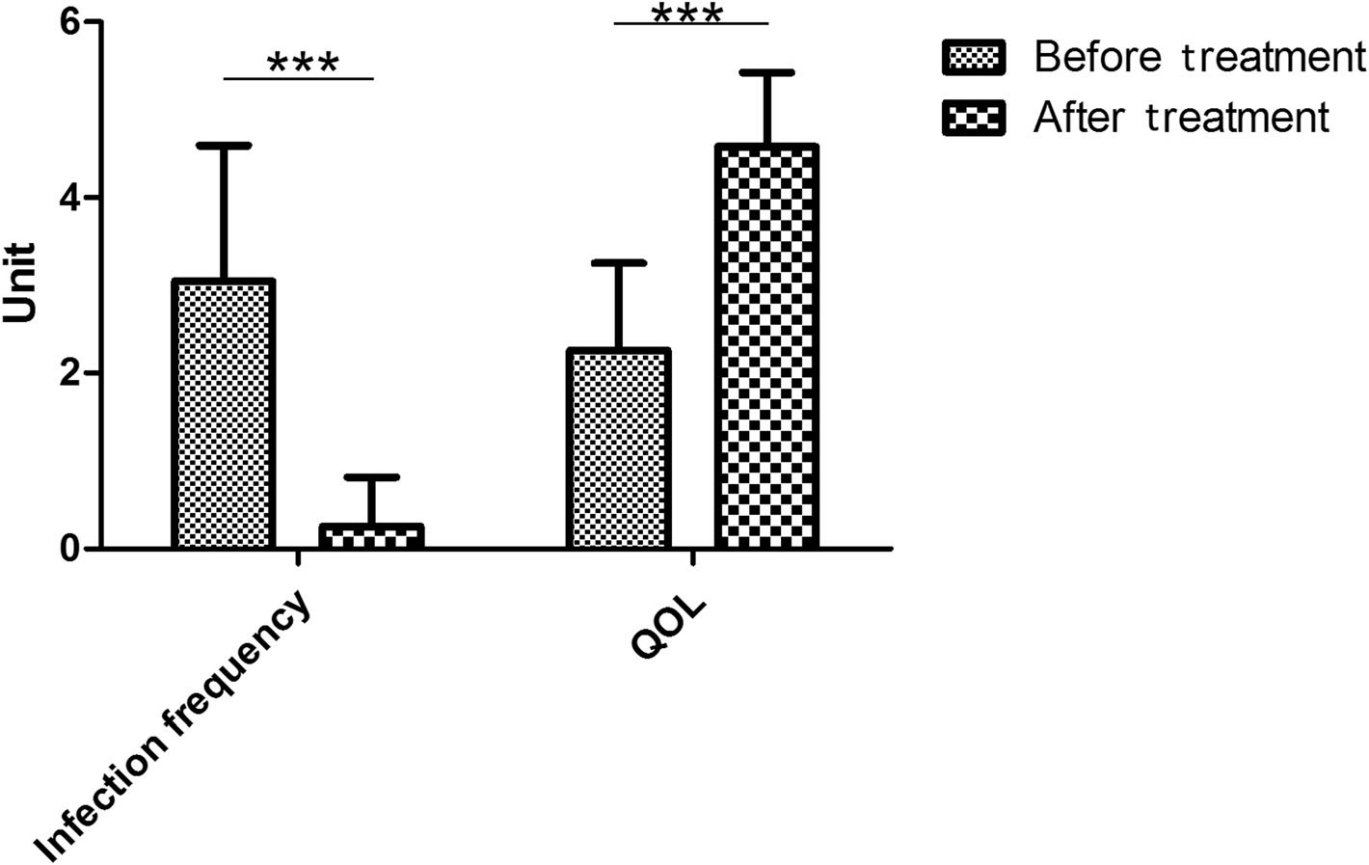
The diagram showed the decreasing of infection frequency and improvement of quality of life (QOL). ***p < 0.001

Before treatment, patients underwent an episode of infection 3 (range 1–6) times per year and after the treatment, the frequency of infection decreased at 0 (range 0–2) times per year (Table 2) (Figs. 1, 2, and 3).

**Table 2:** Independent variables and scar scores for subjective and objective evaluation

Characteristic	Before treatment [median (range)]	After treatment [median (range)]	*p* value
Infection frequency (time/year)	3 (1.6)	0 (0.2)	< 0.001
Pain (score)	65 (22.90)	45 (21.69)	< 0.001
Itch (score)	56 (21.91)	33 (16.61)	< 0.001
QOL (score)	3 (1.3)	5 (3.5)	< 0.001
Blood supply (unit, mean)	130.48 ± 27.29	112.73 ± 26.92	< 0.001

*QOL* quality of life

**Fig. 4:**
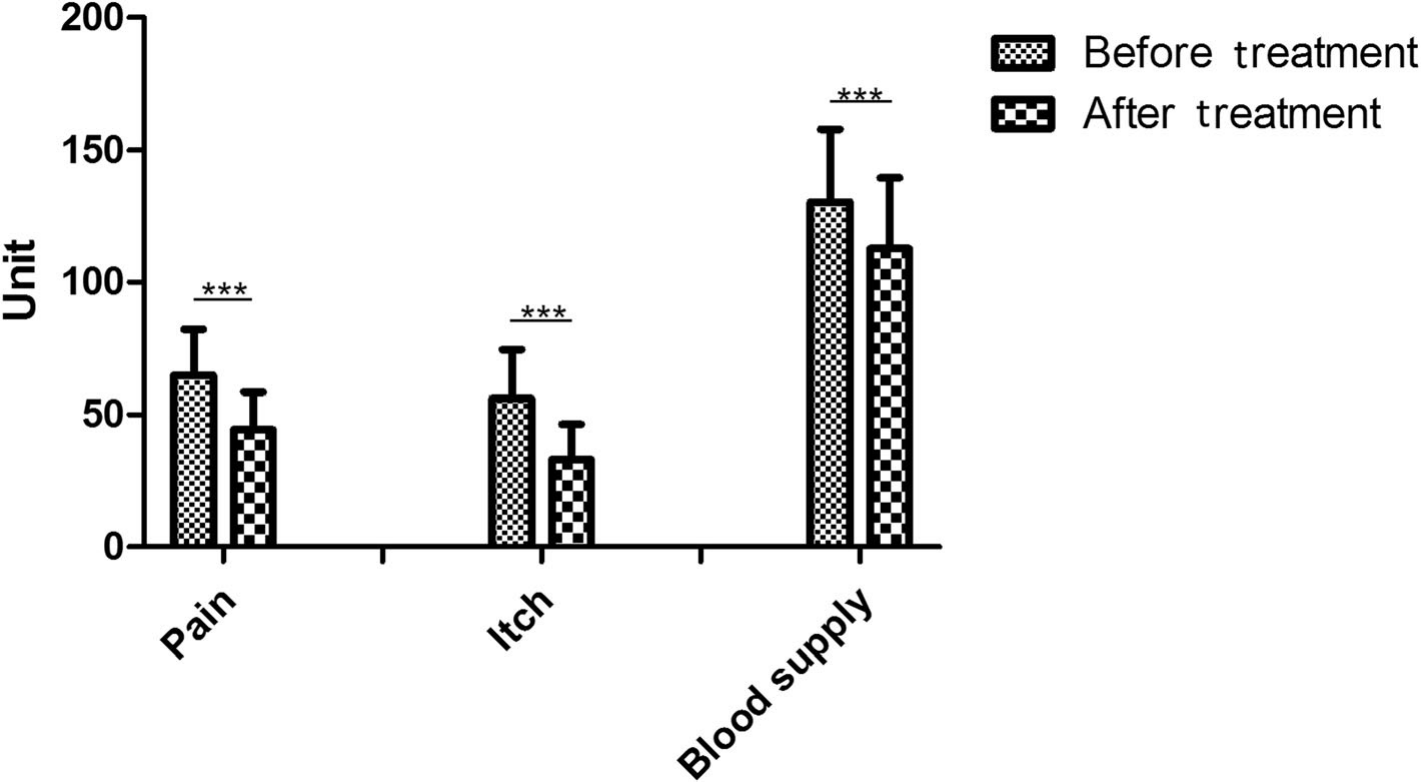
The diagram showed the reduction of pain, itch, and blood supply. ***p < 0.001

Before treatment, the average blood perfusion volume in the scar was 130.48 ± 27.29 unit by using PeriCam PSI blood perfusion imager. After treatment, it was 112.73 ± 26.92 unit (*p* < 0.001). This indicates that the 1470 nm laser can significantly reduce blood flow in the scar (Table 2) (Fig. 4).

### Subjective evaluation

Subjective evaluation measuring pain and itchiness was performed using VAS score. The scores, variations, and outcomes are showed in Fig. 4 and Table 2.

Across the entire cohort, the mean score for impact on QOL before operation was 3 (range 1–3) (0 = profound limitation, 5 = no limitation). The impact of the scar upon a patient’s QOL increased significantly after treatment 5 (range 3–5) (*p* < 0.001) (Fig. 3).

## Discussion

In recent years, several therapeutic options for the treatment of keloid scars have been proposed. Silicone gel sheeting, chemotherapy agents (5-FU, bleomycin) or corticosteroids injections, and radiation therapy (12–20 Gy in five doses) have been used with a range of success, but the risks have made some clinicians avoid it altogether [6–8, 21, 22]. Recently, laser treatment for scars has received significant attention due to its promising results. Several treatment protocols and the use of different laser wavelengths have been proposed, and anecdotal evidence supports their use [21–24]. The author’s experience, in using the 1470 nm diode laser with intralesional fiber to treat inflamed or infected keloids, is reported in this study.

The first described use of a laser for scar treatment was in 1978 adopting a continuous wave argon laser to treat hypertrophic scars and keloids [18]. Despite encouraging early reports, following studies showed limited efficacy and raised incidence of side effects [18]. Castro used Nd:YAG (1064 nm) laser in 1983, and other authors used the continuous wave CO_2_ laser (10,600 nm) in the early 1980s to selectively prevent collagen production [12, 13, 18]. However, early outcomes were poor and further studies demonstrated that keloid formation was not inhibited after 1 year. With the introduction of the theory of selective photothermolysis in the early 1980s, pulsed lasers were developed to provide target selectivity and limit thermal injury and scarring [19]. Development in the early 2000s saw the use of fractionated lasers working by thermally altering a fraction of the targeted site, and due to their features permitting rapid epidermal tissue repair, leaving up to 95% of skin intact which repopulates the ablated columns.

We used the LASEmaR® 1500, a fractional non-ablative laser with 1470 nm wavelength. The more recently introduced a 1470 nm wavelength laser fiber uses a diode output to emit infrared invisible light at wavelengths of 1470 nm, which are absorbed mainly by water and deoxyhemoglobin, producing direct thermal damage on the endothelial cells, suppressing angiogenesis and endothelial cell growth factors and causing vessels ablation. The 1470 nm wavelength fiber has the ability to penetrate through the tissue to a depth of 2–3 mm using a directional laser irradiation. The heat produced from this laser is emitted in a narrow field, so water quickly vaporizes in the surrounding tissues, resulting in selected tissue ablation and coagulation, with localized cell lysis and tissue necrosis.

Gayen et al. used the 1470 nm fiber laser to weld* in vitro* tissue in 2003 [13]. In 2007, Seitz used the 1470 nm fiber laser for the first time in clinical practice to treat benign prostatic hyperplasia [10]. More recently, the 1470 nm laser is used in the treatment of a variety of clinical disorders including respiratory tract problems due to nasal septum deviation or inferior turbinate hypertrophy [14], varicosity [15], prostatic hyperplasia [10], excessive fat deposition [16], and anorectal fistula [17].

Some researchers had proven that the proliferation of fibroblasts in keloids increased significantly, of which the apoptosis ratio decreased. Also, in keloids, epidermal stem cells are deficient. Myofibroblasts got excessive proliferation [20], and the number of transforming growth factor-beta (TGF-β) receptors 1increased significantly [6]. These fibroblasts secrete several cytokines stimulating collagen synthesis and inhibiting collagen decomposition, such as TGF-β, platelet-derived growth factor, and matrix metalloproteinases [18, 21]. The cytokines increase collagen synthesis ability in keloids 6 to 20 times more than normal healthy skin and fibronectin synthetic ability four times more than normal intact skin. All of the above factors resulted in scar thickness significantly increasing.

Our research showed that 1470 nm laser can effectively reduce the inflammation and episodes of infection (*p* < 0.001), thereby suppressing its deterioration. The episodes of infection before treatment were 3.05± 1.54 times per year, and after the treatment the frequency of infection was almost 0.26± 0.56 times per year.

Other authors have proved that the proliferation of fibroblasts in keloids is enhanced, increasing collagen synthesis [6, 25, 26] and cell activities and metabolism promoting new capillaries to transport nutrients. Ogawa et al. [27–29] noted that angiogenic cytokine, vascular endothelial growth factor levels, and vascular density in hypertrophic scars and keloids were significantly higher than normal tissue, assuming that the blood supply is the most important factor for the scar tissue nutrition. As demonstrated by Seitz et al. the 1470 nm wavelength has important properties to be absorbed by hemoglobin, especially deoxyhemoglobin resulting in blood vessel ablation [11, 12]. We have applied this principle to treat inflamed keloids, and we found that after 1 year of laser treatment, the scar blood perfusion decreased as shown in Table 2 by the PeriCam PSI blood perfusion imager (*p* < 0.001). Therefore, we believe that the 1470 nm laser fiber is able to target the hemoglobin and surrounding blood vessels releasing adequate thermal effect to reduce the nutritional supply to the scar and prevent scar tissue.

According to VAS subjective assessment, the itching and pain were decreased (*p* < 0.001). The results showed that 1470 nm fiber can also improve the QOL of patients affected by inflamed keloids (Figs. 3 and 4).

Although the reduction of infections occurrence after 1470 nm fiber on keloids is remarkable, there are still few limitations of this procedure. In particular, first, the 1470 nm fiber laser therapy needs more than one treatment, usually two or three to achieve a satisfactory effect in term of blood supply reduction. Then, the 1470 nm laser can be combined with other laser to inhibit scar hyperplasia or prevent late recurrence [30, 31].

## Conclusion

The present study shows that 1470 nm fiber laser treatment improved inflamed keloids fairly well. The mechanism of improvement after 1470 nm laser treatment therefore likely includes the removal of part of the fibrotic scar, reduction of nutrition vessels, decreasing inflammation, and a relative stabilization of collagen composition. The 1470 nm laser can significantly decrease episodes of infections, itching, and pain, thus improve QOL. Therefore, it is an effective minimally invasive scar therapy, but further studies with more subjects and long-term follow-up are essential to confirm the present results. Furthermore, investigating fiber laser therapy, in combination with other laser devices and applications can enrich the field with alternative treatments.

## Abbreviations

QOL: Quality of life
VAS: Visual analogue scale
